# Recent advancements in iodide/phosphine-mediated photoredox radical reactions

**DOI:** 10.3762/bjoc.19.131

**Published:** 2023-11-22

**Authors:** Tinglan Liu, Yu Zhou, Junhong Tang, Chengming Wang

**Affiliations:** 1 Department of Chemistry, Jinan University, Guangzhou 511443, P. R. China; 2 UNITEST, Weifang 261000, P. R. China

**Keywords:** annulation, decarboxylative, iodide/phosphine, photocatalytic, radical reaction

## Abstract

Photoredox catalysis plays a crucial role in contemporary synthetic organic chemistry. Since the groundbreaking work of Shang and Fu on photocatalytic decarboxylative alkylations in 2019, a wide range of organic transformations, such as alkylation, alkenylation, cyclization, amination, iodination, and monofluoromethylation, have been progressively achieved using a combination of iodide and PPh_3_. In this review, we primarily focus on summarizing the recent advancements in inexpensive and readily available iodide/phosphine-mediated photoredox radical transformations.

## Introduction

Over the past few decades, numerous remarkable breakthroughs and notable progresses have been achieved in the realm of photoredox catalysis [1–3]. This domain has profoundly transformed modern organic synthesis, resulting in a considerable surge in research efforts centered on free radical reactions [4]. Presently, photoredox catalysis has risen to prominence as an incredibly effective methodology, establishing itself as a powerful tool for crafting various C–X (X = C, N, O, F, Cl…) bonds owing to its advantageous traits, such as sustainability, practicality, and environmental compatibility [5].

Despite its broad synthetic utilities, there are still a few drawbacks associated with these photoredox reactions. One of the main limitations is the reliance on precious metals such as Ir, Ru, and Pd, or elaborate organic dyes that act as photosensitizers, which are either limited in abundance or require additional synthetic steps to obtain, thus greatly impeding the widespread application of photoredox catalysis in large-scale industrial processes.

**Scheme 1:**
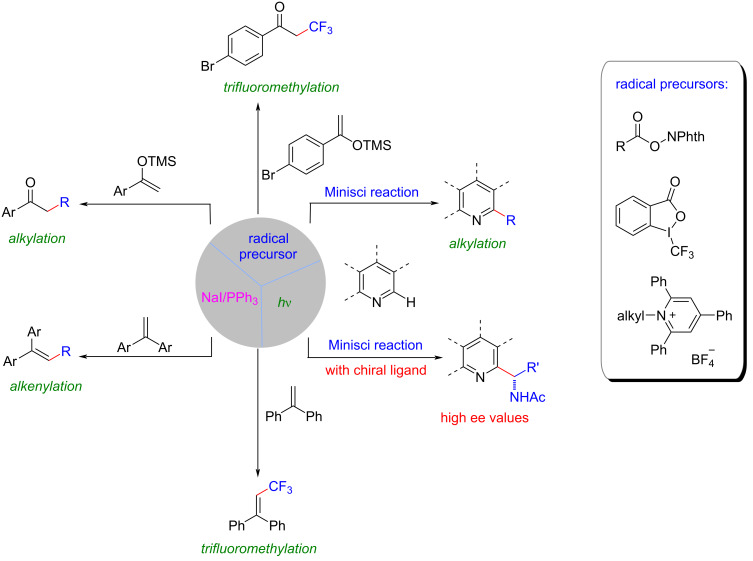
Photocatalytic decarboxylative transformations mediated by the NaI/PPh_3_ catalyst system.

In this context, in 2019, Shang, Fu, and their colleagues made an important breakthrough in addressing these above-mentioned limitations [6]. They disclosed a photocatalytic decarboxylative alkylation reaction that was facilitated by the synergistic action of a cost-effective and easily accessible NaI/PPh_3_ catalyst system (Scheme 1). This system offered an alternative to the use of precious metals or complex organic dyes as catalysts. The developed NaI/PPh_3_-based system not only provided a more sustainable and economically viable approach but also demonstrated excellent performance in various transformations. It had been successfully applied to a series of radical reactions, including trifluoromethylation, deaminative alkylation, and asymmetric versions of Minisci reactions, resulting in good to excellent yields and enantioselectivity. This groundbreaking work opened up new possibilities for the practical application of photoredox catalysis in large-scale industrial processes, as it provided a more accessible and cost-effective catalyst system that could be readily utilized for a wide range of transformations [7–8].

**Scheme 2:**
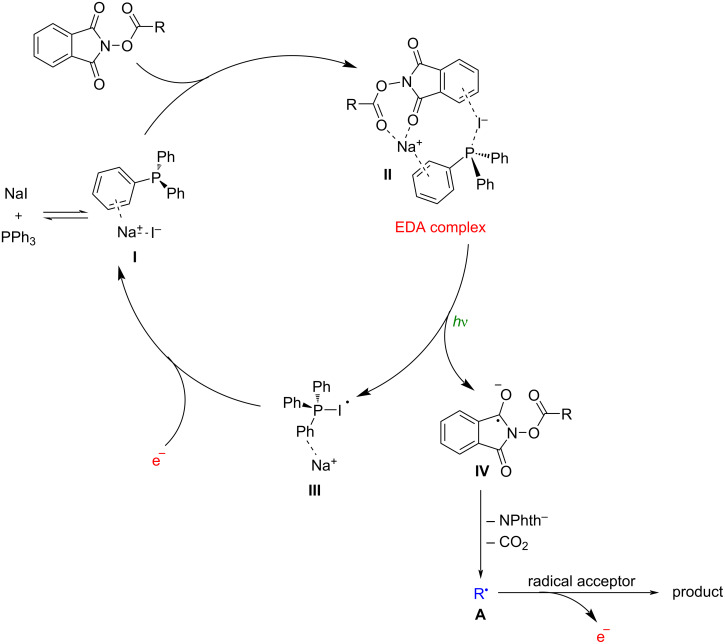
Proposed catalytic cycle of NaI/PPh_3_ photoredox catalysis.

Moreover, they proposed a plausible mechanism for the aforementioned conversions (Scheme 2). Initially, an NaI/PPh_3_ complex **I** was formed through a cation–π interaction. Subsequently, the combination of complex **I** with *N*-(cyclohexanecarbonyloxy)phthalimide smoothly delivered an electron donor–acceptor (EDA) complex **II** via coulombic interactions. Upon 456 nm blue LED light irradiation, the EDA complex **II** underwent a single electron transfer (SET) process, followed by subsequent decarboxylation to produce the alkyl radical intermediate **A**, accompanied by electron release. The radical intermediate **A** could then be captured by a series of different radical acceptors. Finally, the initial NaI/PPh_3_ complex **I** was regenerated from complex **III** through an electron injection/reduction process.

This article aims to provide a comprehensive overview of the latest advancements in the iodide/phosphine catalytic photoredox system. The primary focus of the paper is to delve into the unique catalytic reactivity exhibited by the iodide/phosphine photoredox system, while also exploring potential reaction mechanisms. It is mainly organized around different types of reactions, providing a structured and systematic analysis of each category.

## Review

### Iodide/phosphine-catalyzed photoredox transformations

Since the seminal work of Shang and Fu, the established NaI/PPh_3_ combined system has paved the way for a wide range of photoredox reactions. These reactions encompass diverse transformations such as alkenylation, alkylation, cyclization, amination, iodination, and many others. The discovery of these conversions has significantly expanded the scope and versatility of the NaI/PPh_3_ catalytic system, now making it a powerful tool in synthesis.

#### Alkenylation

**Scheme 3:**
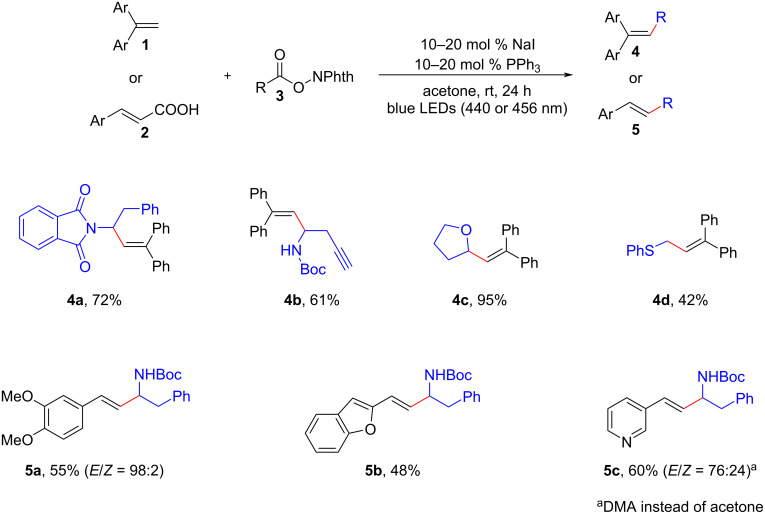
Decarboxylative alkenylation of redox-active esters by NaI/PPh_3_ catalysis.

In 2020, Shang, Fu, and colleagues reported on the photocatalytic decarboxylative alkenylation reactions facilitated by cooperative NaI/PPh_3_ catalysis [9]. These conversions involved the coupling of 1,1-diarylethene/cinnamic acid derivatives (**1**, **2**) with redox-active esters **3** (Scheme 3). Notably, the reactions were driven by blue light irradiation at either 440 nm or 456 nm, and they occurred in acetone at room temperature, without the need for transition metals or organic dyes as photosensitizers. Interestingly, it was discovered that solvation played a vital role in the overall process. These findings shed light on the mechanistic aspects of the reaction and highlighted the potential of the NaI/PPh_3_ catalytic system for achieving efficient and transition-metal-free photocatalytic transformations.

**Scheme 4:**
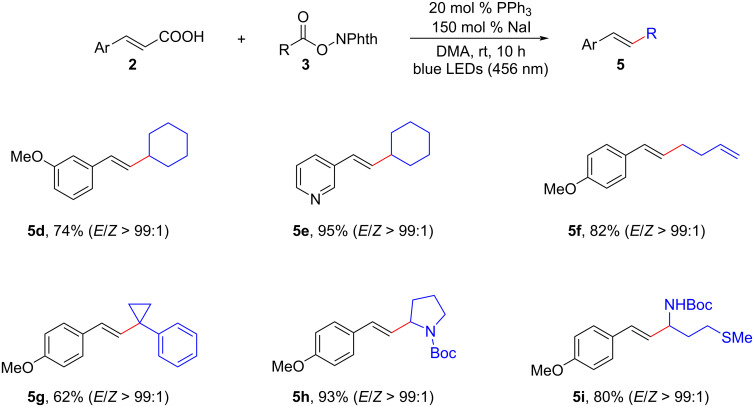
Decarboxylative alkenylation mediated by NaI/PPh_3_ catalysis.

Following that, Li and his research group documented similar results (Scheme 4) [10]. They extensively investigated the compatibility and efficiency of a diverse range of redox-active esters **3**, deriving from various aliphatic carboxylic acids (including primary, secondary, and tertiary acids), as well as α-amino acids. Impressively, these redox-active esters exhibited exceptional compatibility, high effectiveness, and remarkable specificity in the synthesis of β-alkylated styrenes **5**. This study underscored the broad applicability and selectivity of the NaI/PPh_3_ catalytic system in facilitating the synthesis of β-alkylated styrenes using diverse redox-active esters.

**Scheme 5:**
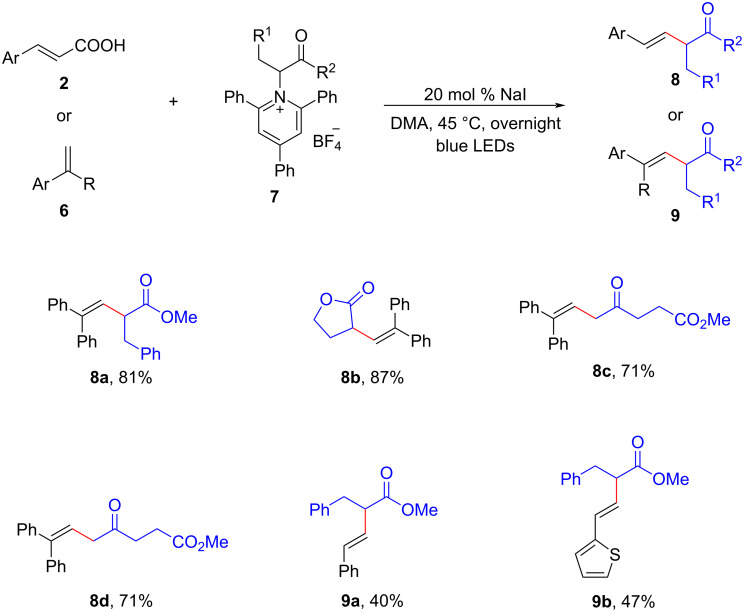
NaI-mediated photoinduced α-alkenylation of Katritzky salts **7**.

It is worth highlighting that triphenylphosphine is not essential for the photoredox cross-coupling reactions discussed above. A recent elegant study conducted by Chen and colleagues introduced a straightforward method that directly employed sodium iodide for photoinduced deaminative alkenylation processes [11]. This method enabled the synthesis of β,γ-unsaturated esters **8**, **9** without the requirements of phosphine or other photocatalysts (Scheme 5). Through the use of density functional theory (DFT) calculations, they elucidated the mechanism behind this process. It was revealed that the formation of a photoactive EDA complex, which subsequently generated alkyl radicals for alkenylation, was primarily facilitated by the electrostatic interaction between NaI and Katritzky salts **7**. This innovative approach not only expanded the scope of photoredox cross-coupling reactions but also offered valuable insights into the role of NaI in facilitating these transformations.

**Scheme 6:**
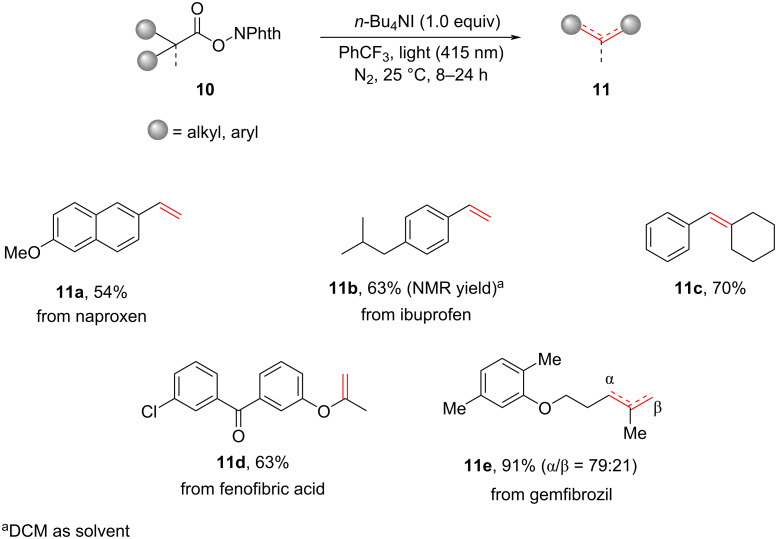
*n*-Bu_4_NI-mediated photoinduced decarboxylative olefination.

In a recent study, Zheng et al*.* introduced a highly effective photocatalytic approach for the decarboxylative conversion of redox-active esters **10**, leading to the efficient synthesis of olefins **11**. This process was conducted in the presence of *n*-Bu_4_NI, as illustrated in Scheme 6 [12]. The utilization of mild reaction conditions allowed for the application of this method in the modification of complex natural products or pharmaceuticals. Moreover, this photoinduced decarboxylative approach demonstrated the potential for broader utilization in the construction of diverse C(sp^3^)–N and C(sp^3^)–X bonds.

**Scheme 7:**
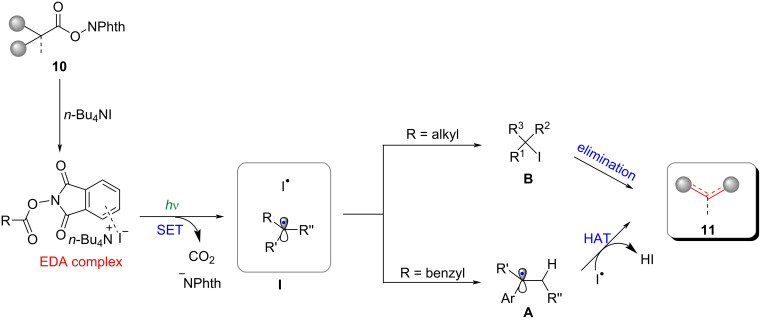
Proposed mechanism of the *n*-Bu_4_NI-mediated photoinduced decarboxylative olefination.

An EDA complex was formed through non-covalent interaction between the redox ester **10** and *n*-Bu_4_NI (Scheme 7). Subsequently, upon the photoexcitation, radical pairs **I** were generated via a SET process, accompanied by the liberation of CO_2_ and the phthalimide anion. The recombination of the alkyl radical and **I^·^** played a pivotal as an intermediate step in the production of alkyl iodides **B**. Compound **B** could undergo a further elimination reaction to yield various olefins **11**.

Regarding benzyl substrates, the radical **I^·^** demonstrated its efficacy as a reagent for hydrogen atom transfer (HAT), specifically by extracting a hydrogen atom from the α-position of benzyl radicals **A**. The process described above led to the formation of the corresponding olefins **11**, eliminating the need for a carbon–iodine bond formation step.

#### Alkylation

**Scheme 8:**
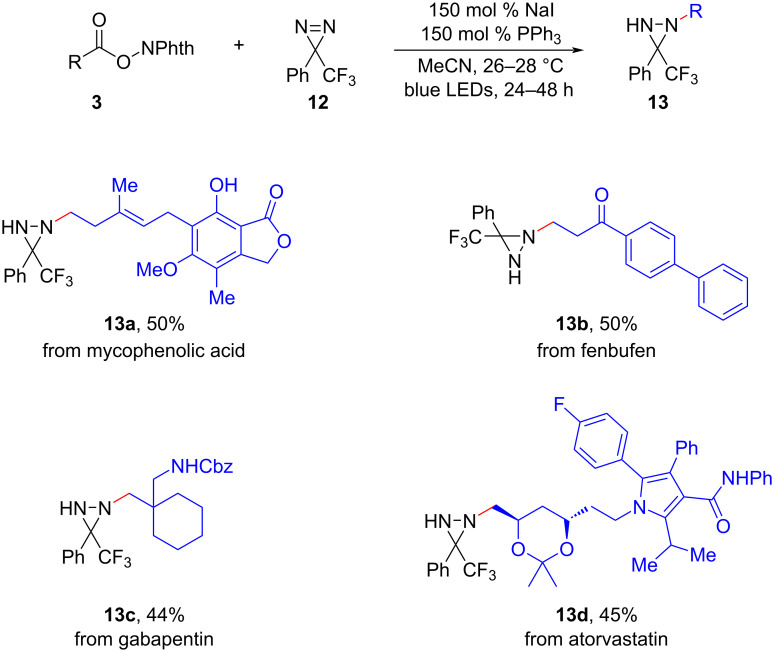
Photodecarboxylative alkylation of redox-active esters with diazirines.

Diaziridines are highly versatile building blocks in synthesis, with the ability to be readily transformed into various valuable functional molecules, including amines, hydrazines, and nitrogen-containing heterocycles [13]. In a significant advancement in 2021, Lopchuk et al. revealed a novel method for the photodecarboxylative alkylation of diazirines **12** using the readily accessible redox-active esters **3** and cost-effective NaI/PPh_3_ photoactivators under mild reaction conditions (Scheme 8) [14]. The methodology exhibited remarkable efficacy when applied to a wide range of natural products and pharmaceuticals, significantly expanding the synthetic utility of this approach. Importantly, the demonstration of the exceptional compatibility between blue LEDs and diazirine compounds also held the promise of inspiring further exploration and development of novel synthetic strategies in this field.

**Scheme 9:**
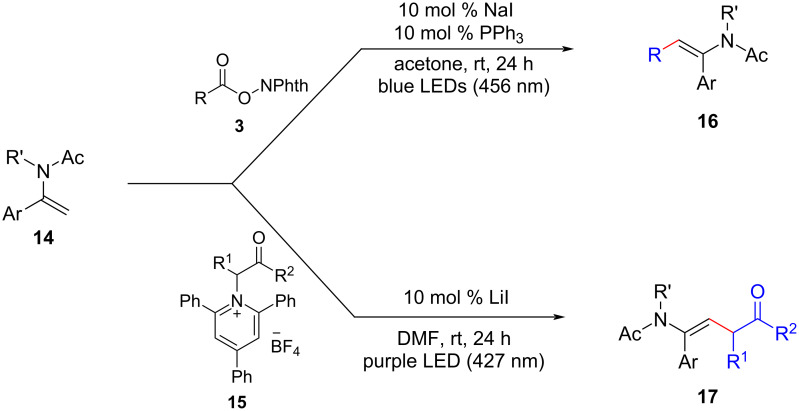
Photoinduced iodine-anion-catalyzed decarboxylative/deaminative C–H alkylation of enamides.

Enamides are commonly found in medicinal compounds and physiologically active natural products. The direct functionalization of C–H bonds in enamides offers a convenient and versatile approach to access a wide range of functionalized enamides. In 2021, Fu and his colleagues successfully developed a novel method for the stereoselective alkylation of enamides **14** using iodine-anion catalysis under visible light irradiation, as depicted in Scheme 9. Subsequent investigations revealed that redox-active esters **3** and Katritzky salts **15** derived from amino acids could be effectively employed in decarboxylative/deaminative cross-coupling reactions [15]. These reactions enabled the efficient synthesis of diversely functionalized enamides **16** and **17**, demonstrating remarkable tolerance towards various functional groups.

**Scheme 10:**
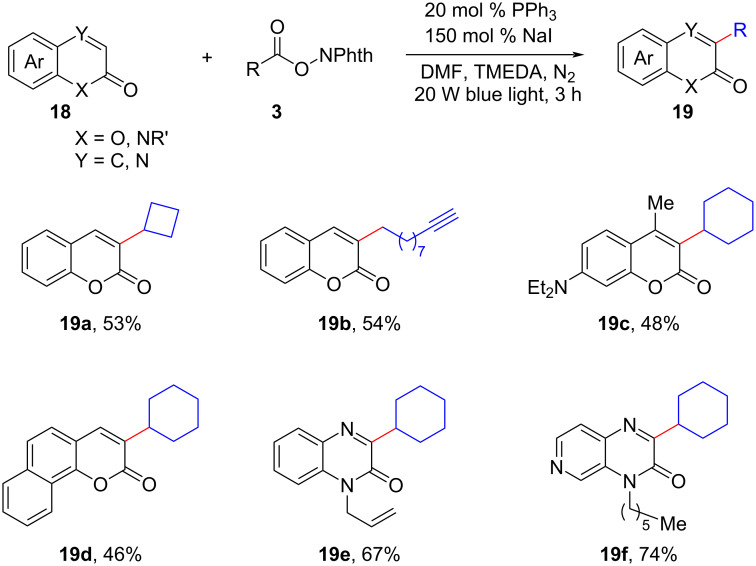
Photocatalytic C–H alkylation of coumarins mediated by NaI/PPh_3_ catalysis.

In recent years, there has been a surge of research interest in coumarin derivatives due to their notable biological, pharmacological, and optical properties [16]. Zhou and colleagues introduced an interesting metal- and oxidant-free photocatalytic C–H alkylation method for coumarins **18** [17]. The method utilized triphenylphosphine and sodium iodide, along with readily available alkyl *N*-hydroxyphthalimide esters (NHPIs) **3** as the alkylation reagents (Scheme 10). Impressively, this transformation exhibited exceptional versatility, extending beyond coumarins to encompass other nitrogen-containing heterocycles, including quinoxalinones, with remarkable C-3 regioselectivity. The findings of this study significantly expanded the synthetic toolbox for accessing functionalized coumarin derivatives and related nitrogen-containing heterocycles, opening up exciting possibilities for their diverse applications in other fields. Similarly, the regioselective photodecarboxylative C–H alkylation of 2*H*-indazoles and azauracils using NaI/PPh_3_ as mediators and redox esters **3** was reported by the research groups of Murarka [18] and Fan [19], respectively.

**Scheme 11:**
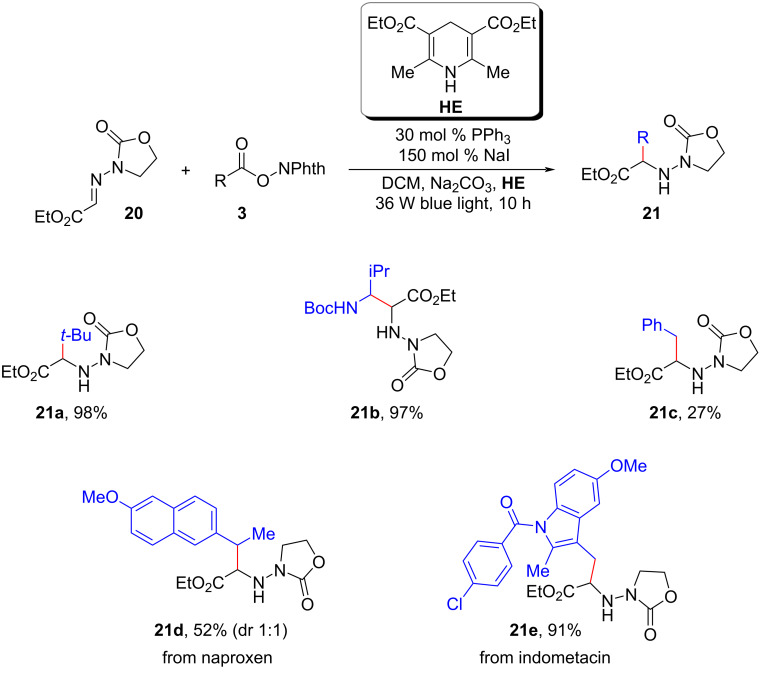
Photoredox alkylation of aldimines by NaI/PPh_3_ catalysis.

Simultaneously, Shen and colleagues made a notable contribution by disclosing a NaI/PPh_3_ EDA complex-mediated photoredox alkylation of aldimines **20** (Scheme 11) [20]. This newly developed method offered a reliable and efficient route for the synthesis of unnatural amino acids and amines. Remarkably, the procedure exhibited excellent compatibility with a wide range of alkyl radicals, including primary, secondary, tertiary, and α-heterosubstituted radicals generated from corresponding redox-active esters **3**.

**Scheme 12:**
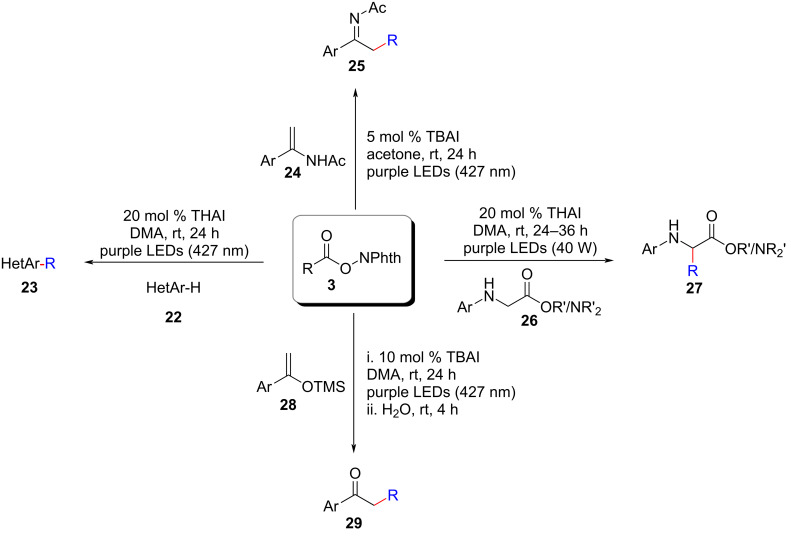
Photoredox C–H alkylation employing ammonium iodide.

Concurrently, Shang and colleagues achieved a significant breakthrough by sequentially unveiling a series of decarboxylative alkylation reactions involving heteroarenes **22**, enamides **24**, *N*-arylglycine derivatives **26**, and silyl enol ethers **28** [21–22]. Notably, these transformations were accomplished using only a catalytic amount of ammonium iodide under irradiation in the absence of triphenylphosphine (Scheme 12). The generation of alkyl radicals was attributed to the photoactivation of a transient electron donor–acceptor complex formed between iodide and *N*-(acyloxy)phthalimide, in line with earlier findings. These remarkable advances not only highlighted the synthetic potential of photocatalysis but also served as inspiration for future developments of low-cost photocatalysis based on other non-covalent interactions. The simplicity, practicality, and broad substrate scope demonstrated by these approaches further emphasized their significance in facilitating the synthesis of diverse compounds and paving the way for further advancements in the field of photocatalysis.

**Scheme 13:**
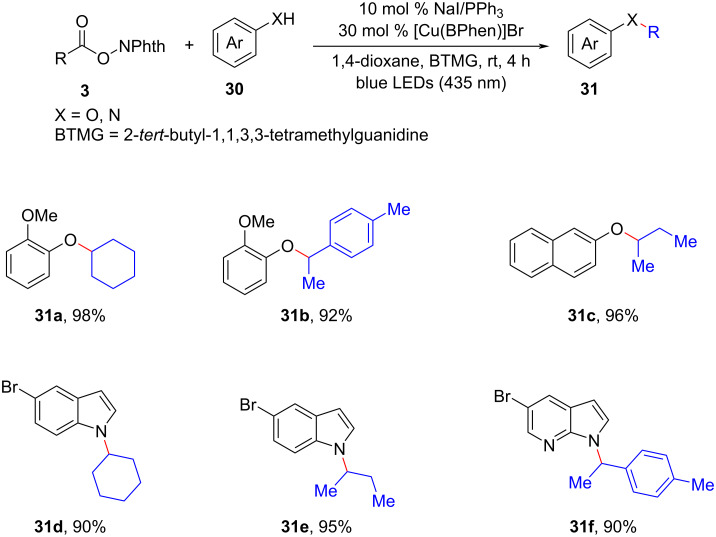
NaI/PPh_3_/CuBr cooperative catalysis for photocatalytic C(sp^3^)–O/N cross-coupling reactions.

The highly efficient construction of carbon–heteroatom (C–X) bonds is of significant importance in the fields of natural products, pharmaceuticals, and materials science. In recent years, the combination of dual photoredox with first-row transition-metal catalysis has emerged as a powerful tool for achieving various cross-coupling reactions involving C–N, C–O, C–S, and other chemical bonds [3,23]. In this context, Guan et al. theoretically designed a novel metallaphotoredox catalysis by combining the NaI/PPh_3_ photoredox catalyst with a Cu(I) catalyst to accomplish diverse C–O/N cross-couplings of alkyl *N*-hydroxyphthalimide esters **3** with various phenols/secondary amines **30** (Scheme 13) [24]. It was anticipated the utilization of computational methods in organic synthesis would provide new insights and novel concepts for the exploration of other metallaphotoredox catalytic systems, thus greatly speeding up the process of new reaction findings.

**Scheme 14:**
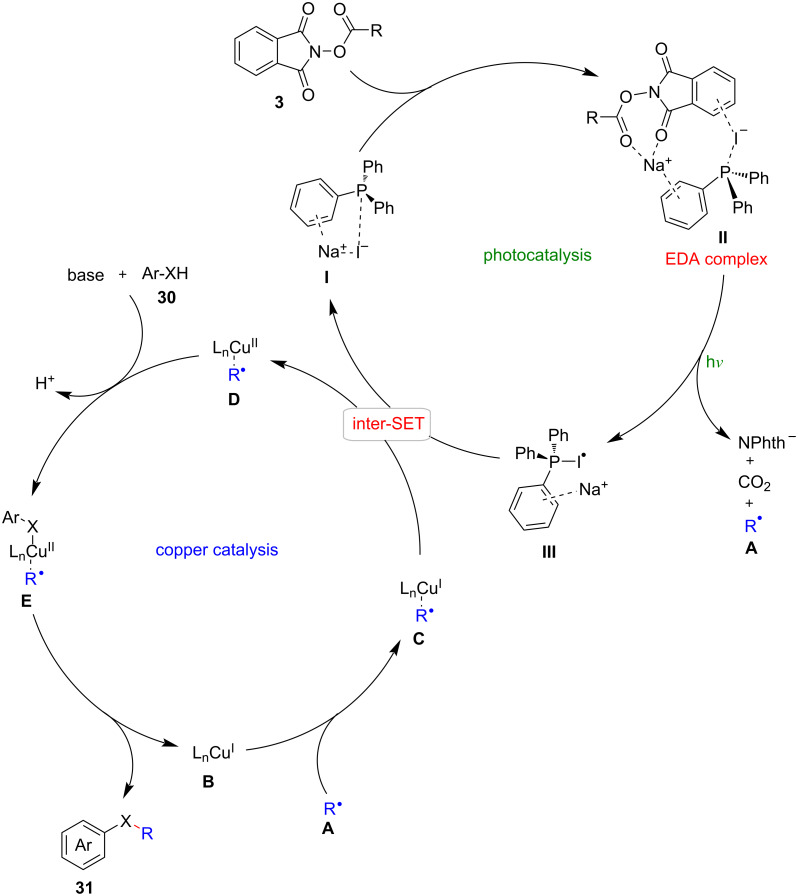
Proposed mechanism of NaI/PPh_3_/CuBr cooperative catalysis for photocatalytic C(sp^3^)–O/N cross-couplings.

An elegant NaI/PPh_3_/CuBr metallaphotoredox dual-catalytic system was responsible for the aforementioned transformations, as depicted in Scheme 14. The dual-catalytic cycle comprised a photocatalytic cycle and a copper catalytic cycle, interconnected through an intermolecular single-electron transfer.

Within the context of the photocatalytic cycle, the generation of the C(sp^3^)-centered alkyl radical **A** was facilitated by the process of photoexcited radical decarboxylation. On the other hand, the copper catalytic cycle involved the capture of alkyl radicals by the copper complex **B**, the activation of heteroatom-containing substrates **30** by a base-mediated proton transfer, and the subsequent reductive elimination process. This reductive elimination led to the formation of C(sp^3^)–X (X = O or N) cross-coupling products **31**.

#### Cyclization

Radical-involved selective C–H functionalizations [25–26], particularly annulation reactions [26], have emerged as highly effective and powerful techniques in synthesis, possessing notable advantages in terms of both step- and atom-economy.

**Scheme 15:**
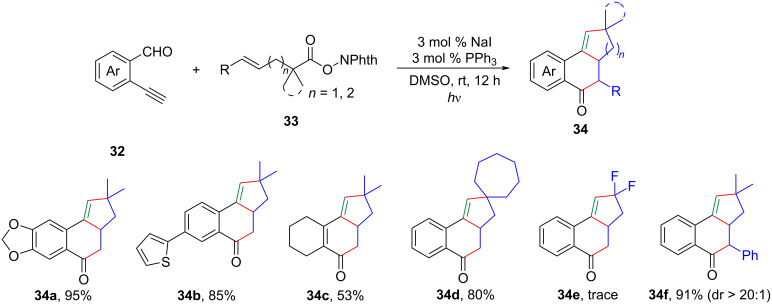
Photocatalytic decarboxylative [3 + 2]/[4 + 2] annulation between enynals and γ,σ-unsaturated *N*-(acyloxy)phthalimides.

Taking inspiration from the groundbreaking work of Shang and Fu [6], Li and colleagues demonstrated an innovative approach for the photocatalytic [3 + 2] and [4 + 2] annulation of enynals **32** and γ,σ-unsaturated *N*-(acyloxy)phthalimides **33** (Scheme 15) [27]. This method involved a series of steps, including the formation of an EDA complex, decarboxylation, radical addition, C–H functionalization, and annulation. Various primary, secondary, and tertiary alkyl *N*-hydroxyphthalimide esters **33** showed potential as viable substrates for the synthesis of fused ketones **34**, eliminating the need for transition-metal catalysts or oxidants. The technique offered a broad substrate scope, remarkable selectivity, and simple reaction conditions.

**Scheme 16:**
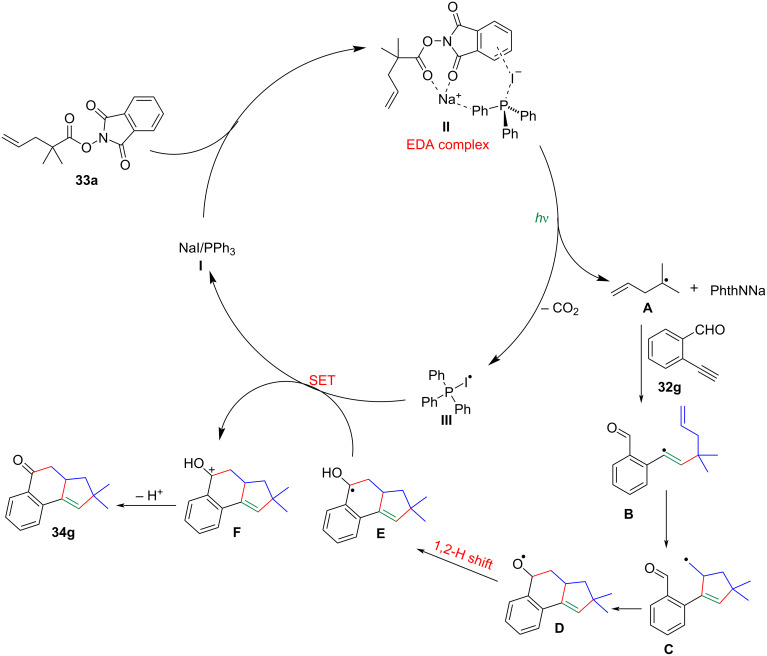
Proposed mechanism for the decarboxylative [3 + 2]/[4 + 2] annulation.

A plausible mechanism had been proposed for the photocatalytic decarboxylative [3 + 2]/[4 + 2] annulation, as depicted in Scheme 16. Initially, a photoactive EDA complex **II** was transiently formed through the combined action of NaI, PPh_3_, and the γ,σ-unsaturated phthalimide **33a**. Upon irradiation with blue LEDs, the alkyl radical **A** was generated through a single-electron transfer from the iodide anion to the γ,σ-unsaturated phthalimide **33a**. Simultaneously, radical **III** of the catalyst was also formed, accompanied by the extrusion of CO_2_. Subsequently, the alkyl radical **A** added to the carbon–carbon triple bond of enynal **32g**, resulting in the formation of a vinyl radical intermediate **B**, followed by a 5-*exo*-*trig* cyclization to release an active alkyl radical intermediate **C**. Once formed, **C** added to the aldehyde group via a [4 + 2] annulation, releasing the alkoxy radical intermediate **D**. The latter then underwent a subsequent 1,2-H atom shift to generate the alkyl radical intermediate **E** which was further oxidized by the Ph_3_P−I^•^ species **III**, forming the cationic intermediate **F**. Finally, deprotonation of intermediate **F** yielded the product **34g**.

Functional polycyclic compounds, such as indene-containing polycyclic motifs and *N*-containing polyheterocycles are commonly found in many natural products and pharmaceuticals, demonstrating significant potential in combating human immunodeficiency virus infections and cardiovascular disorders. The acquisition of these significant structures has predominantly been carried out through a sequential process. Over the decades, chemists have made considerable efforts to improve the construction of these scaffolds [28–31], and one of the most efficient approaches is the cascade cyclization strategy [29–31].

**Scheme 17:**
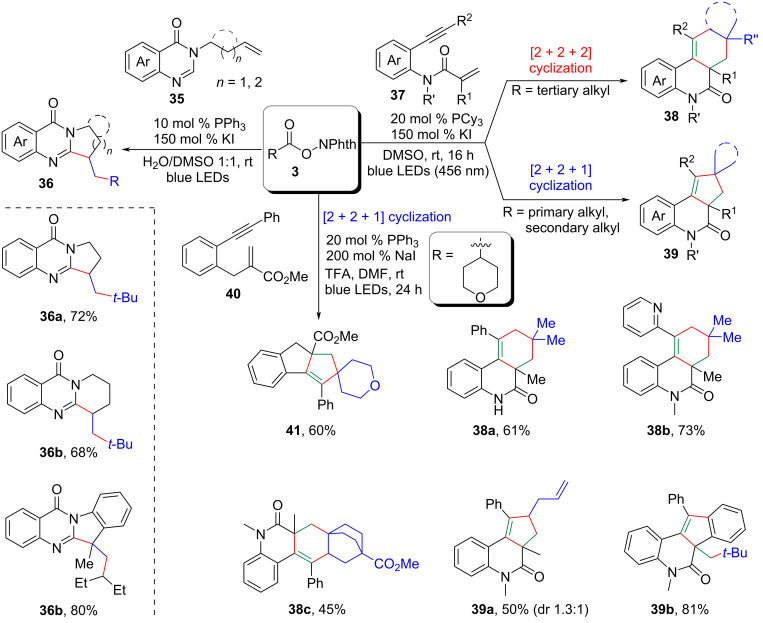
Decarboxylative cascade annulation of alkenes/1,6-enynes with *N*-hydroxyphthalimide esters.

Xu, Li, Wei and their co-workers successfully devised a series of highly regioselective iodide/phosphine synergistically catalyzed photocatalytic cascade annulations for the construction of various nitrogen-containing polycyclic frameworks (**36**, **38**, **39**, **41**) (Scheme 17) [32–34]. These protocols offered a wide range of substrate compatibility in a one-pot reaction, significantly enhancing synthetic efficiency.

Nitrogen-containing heterocycles are abundantly found in nature and represent some of the most prevalent frameworks in natural products, medicines, and functional materials. Despite the development of numerous synthetic methods over the past one century, chemists are still seeking more straightforward routes to access these structurally important and useful *N*-heterocycles.

**Scheme 18:**
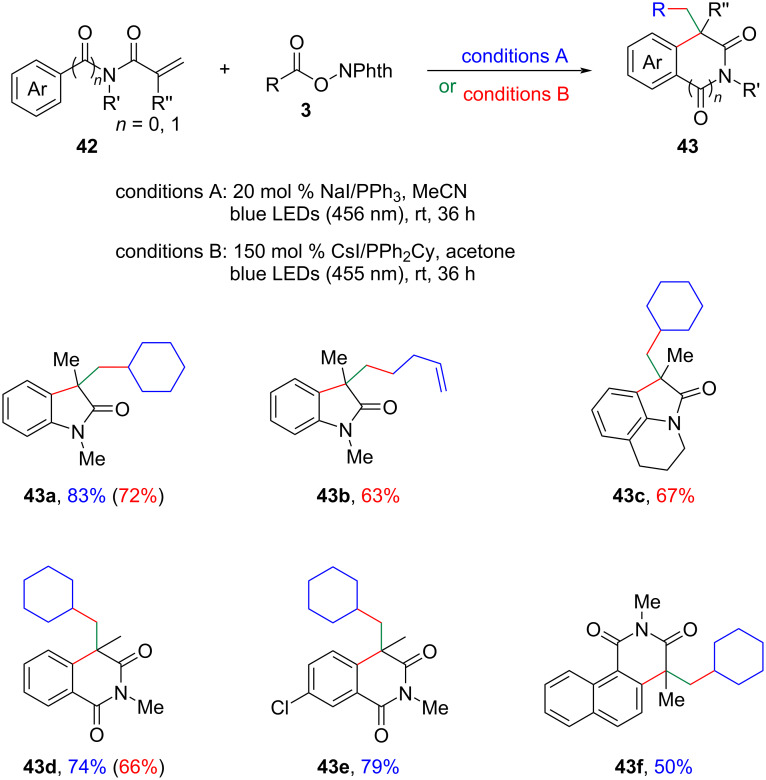
Decarboxylative radical cascade cyclization of *N*-arylacrylamides.

Recently, independent research groups led by Li, Yang, and Patureau separately disclosed a novel approach to 3,3-disubstituted oxindoles **43** through an iodide/phosphine-catalyzed visible-light-mediated decarboxylative radical cascade cyclization of *N*-arylacrylamides **42** (Scheme 18) [35–36]. Importantly, these methodologies could also be smoothly extended to the synthesis of isoquinolinediones, which borne a quaternary carbon center.

**Scheme 19:**
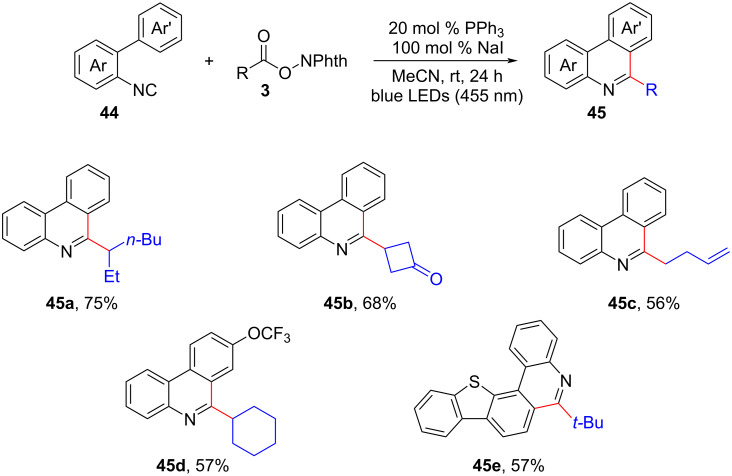
NaI/PPh_3_-driven photocatalytic decarboxylative radical cascade alkylarylation.

Furthermore, Yatham and his colleagues unveiled the first NaI/PPh_3_-mediated photocatalytic decarboxylative cascade cyclization of 2-isocyanobiaryls **44** with alkyl *N*-hydroxyphthalimide esters **3**, resulting in the efficient synthesis of various 6-alkylated phenanthridines **45** (Scheme 19) [37]. The protocol exhibited a wide substrate scope, excellent tolerance towards functional groups, and mild reaction conditions.

**Scheme 20:**
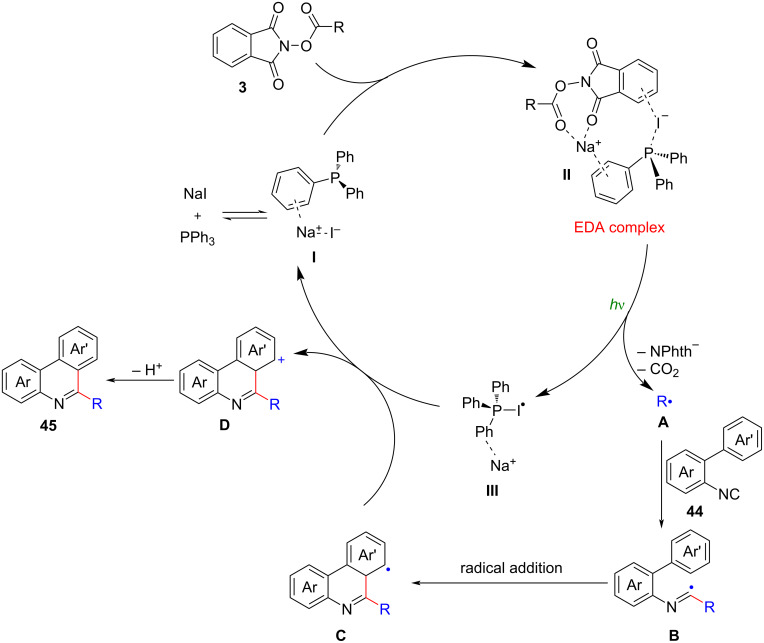
Proposed mechanism of the NaI/PPh_3_-driven photocatalytic decarboxylative radical cascade cyclization.

Based on the experimental observations and a previous report [6], it was proposed that the decarboxylative cascade cyclization reaction proceeded through the formation of a charge-transfer complex (CTC) **II** involving PPh_3_, NaI, and NHP ester **3** (Scheme 20). Upon photofragmentation of the CTC complex **II**, two important intermediates were generated: an alkyl radical **A** and a PPh_3_–I radical **III**. The subsequent isocyanide **44** SOMOphilic insertion reaction led to the formation of an imidoyl radical **B**. This radical then underwent rapid addition onto the C–C double bond, resulting in the release of the desired phenanthridine products **45**. Importantly, this process also replenished the NaI/PPh_3_ catalyst, completing the catalytic cycle.

**Scheme 21:**
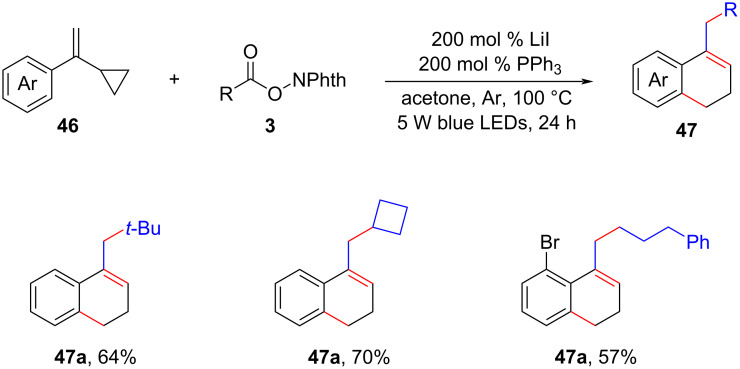
Visible-light-promoted decarboxylative cyclization of vinylcycloalkanes.

Very recently, Zhong and his colleagues proposed a decarboxylative alkylation method for vinylcyclopropanes **46** using alkyl *N*-(acyloxy)phthalimide esters **3**. This methodology enabled the synthesis of variously substituted 2-alkylated 3,4-dihydronaphthalenes **47** with yields of up to 92%, as depicted in Scheme 21 [38]. The key aspect of the approach involved the simultaneous cleavage of dual C–C bonds and a single N–O bond, which was facilitated by the utilization of LiI/PPh_3_ as the photoredox system.

#### Amination

Anilines play important roles in both academic research and industrial applications. As a result, significant efforts have been devoted to the development of various methods for the reduction of nitroarenes [39]. Recent advancements in the catalytic reduction of nitroarenes largely rely on transition-metal catalysis through direct hydrogenation or hydrogen transfer [40], electrocatalysis coupled with water oxidation [41], and sustained visible-light-induced photocatalysis [42]. Among the different strategies available, the use of a mild photocatalytic process involving hole-driven hydrogen transfer with hydrogen donors or hole scavengers has emerged as an attractive approach for nitroarene reduction [43–44].

**Scheme 22:**
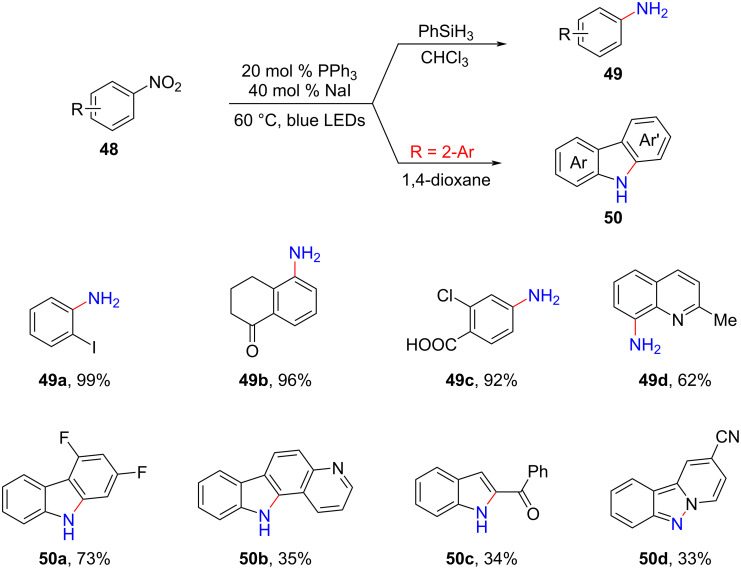
NaI/PPh_3_-mediated photochemical reduction and amination of nitroarenes.

In 2021, Huang and colleagues discovered a photoredox system that did not require any transition metal or other photosensitizers [45]. This system employed a combination of NaI and PPh_3_ to achieve highly selective reduction of nitroarenes **48** (Scheme 22). The protocol demonstrated excellent tolerance towards a wide range of reducible functional groups, including halogens (such as chlorine, bromine, and even iodine), aldehydes, ketones, carboxyl groups, and cyano groups.

#### Iodination

Alkyl iodide is considered to be the most reactive electrophile compared to other alkyl halides, such as related bromides and chlorides. As a result, an effective iododecarboxylation provides a versatile platform for a range of decarboxylative reactions.

**Scheme 23:**
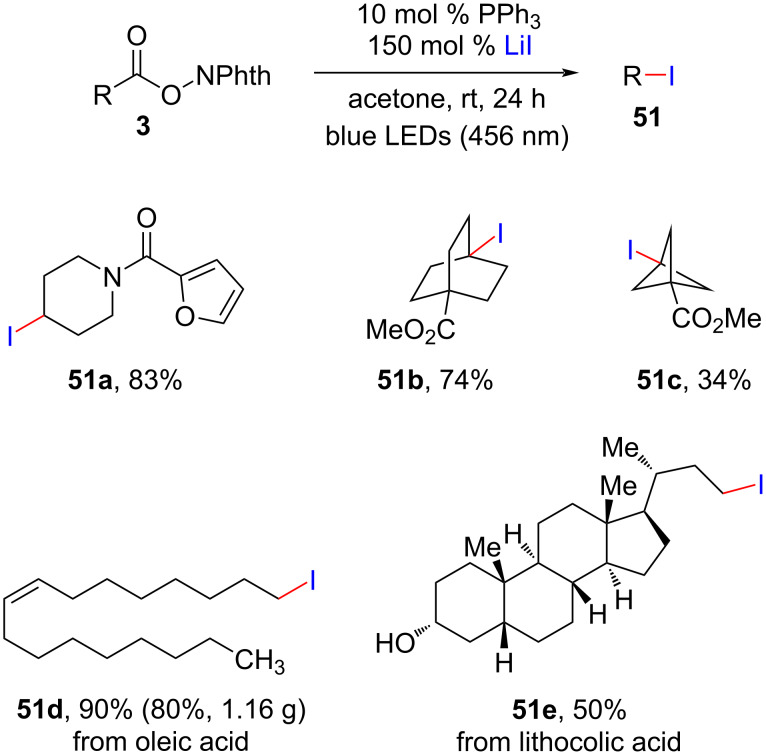
PPh_3_-catalyzed alkylative iododecarboxylation with LiI.

Shang and co-workers recently found that aliphatic carboxylates and lithium iodide could undergo iododecarboxylation under 456 nm blue light irradiation through a PPh_3_-catalyzed procedure (Scheme 23) [46]. Moreover, diversely primary, secondary, tertiary alkyl iodides **51** were easily converted to various C–N, C–O, C–F, and C–S bonds, thus greatly enhancing the potential applications of this chemistry.

**Scheme 24:**
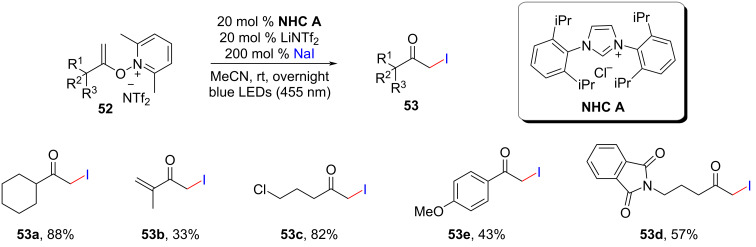
Visible-light-triggered iodination facilitated by N-heterocyclic carbenes.

Meanwhile, the research groups of Chen and Wang demonstrated an elegant use of electrostatic contact to promote radical–radical cross-coupling between *N*-alkenoxypyridinium salts **52** and NaI, resulting in the formation of various α-iodo ketones **53** when exposed to visible light (Scheme 24) [47]. In the process, the NHC catalyst acted as a stabilizer for the EDA complex and generates a radical species, which was confirmed by further computational studies.

#### Monofluoromethylation

The monofluoromethyl (CH_2_F) group, which is commonly found in a lot of agrochemicals, pharmaceuticals, and materials, serves as a powerful bioisostere for a range of functional groups (such as CH_2_OH, CH_2_OCH_3_, CH_2_NH_2_, and CH_2_SH). Among the various methods available, radical-involving cross-couplings have proven to be the most effective [48–49]. However, the generation of the CH_2_F radical remains to be a challenging task. Therefore, there is an urgent need to develop diverse monofluoromethylation methods.

**Scheme 25:**
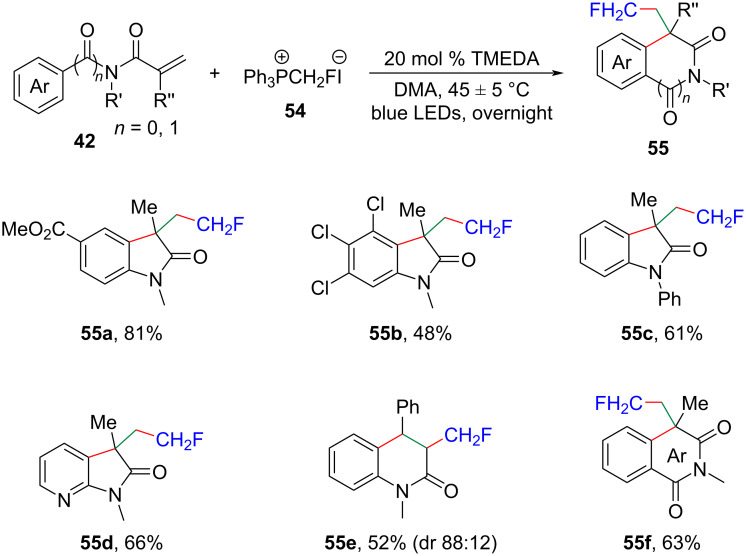
Visible-light-induced photolysis of phosphonium iodide salts for monofluoromethylation.

In this context, Chen and his colleagues recently developed a concise photocatalytic procedure for achieving monofluoromethylation, as well as di- and trifluoromethylation of various alkenes (Scheme 25) [50]. The synthetic method also showcased broad applicability, operational simplicity, and utilized easily obtainable and air-stable phosphonium salts **54** as convenient photoinduced R_f_ radical reagents.

## Conclusion

In recent years, the field of synthetic chemistry has experienced significant advancements in iodide/phosphine-based photoredox radical reactions. These reactions have garnered much attention due to their cost-effectiveness, low toxicity, and widespread availability. Notably, the NaI/PPh_3_ combined system has been successfully employed in the photofixation of nitrogen [51].

Despite these remarkable progresses made, there remain several synthetic challenges that require further investigation and resolution: First, the current reliance on redox-active esters as radical precursors in iodide/phosphine-mediated conversions restricts the potential applications of these conversions in synthesis. Therefore, it is highly recommended to develop other alternative radical precursors, explore new different reaction types (rather than the decarboxylative process), and design novel EDA complexes for photoredox catalysis, in addition to the well-established methods mentioned earlier.

Moreover, asymmetric versions of iodide/phosphine-mediated photoredox radical reactions are relatively scarce [52], representing an unexplored area that requires further investigation. Developing asymmetric methodologies in this domain holds great promise for future exploration.

Last but not the least, conducting detailed mechanistic studies on iodide/phosphine-involved reactions is crucial for gaining a deeper understanding of their underlying mechanisms and expediting the process of designing new reactions.

Overall, addressing these challenges and advancing the field through innovative approaches and mechanistic insights will contribute to the continued progresses and applications of iodide/phosphine-based photoredox radical reactions in synthetic chemistry.

## References

[1] Romero N A, Nicewicz D A (2016). Chem Rev.

[2] Bell J D, Murphy J A (2021). Chem Soc Rev.

[3] Chan A Y, Perry I B, Bissonnette N B, Buksh B F, Edwards G A, Frye L I, Garry O L, Lavagnino M N, Li B X, Liang Y (2022). Chem Rev.

[4] Matsui J K, Lang S B, Heitz D R, Molander G A (2017). ACS Catal.

[5] Shaw M H, Twilton J, MacMillan D W C (2016). J Org Chem.

[6] Fu M-C, Shang R, Zhao B, Wang B, Fu Y (2019). Science.

[7] Noble A, Aggarwal V K (2019). Sci China: Chem.

[8] List B, Li Y (2019). Synfacts.

[9] Wang Y-T, Fu M-C, Zhao B, Shang R, Fu Y (2020). Chem Commun.

[10] Wang H-Y, Zhong L-J, Lv G-F, Li Y, Li J-H (2020). Org Biomol Chem.

[11] Zhang C-S, Bao L, Chen K-Q, Wang Z-X, Chen X-Y (2021). Org Lett.

[12] Luo J-j, Jing D, Lu C, Zheng K (2023). Eur J Org Chem.

[13] Ravindra S, Jesin C P I, Shabashini A, Nandi G C (2021). Adv Synth Catal.

[14] Maharaj V, Chandrachud P P, Che W, Wojtas L, Lopchuk J M (2021). Org Lett.

[15] Wang J-X, Wang Y-T, Zhang H, Fu M-C (2021). Org Chem Front.

[16] Bouhaoui A, Eddahmi M, Dib M, Khouili M, Aires A, Catto M, Bouissane L (2021). ChemistrySelect.

[17] Gan X, Wu S, Geng F, Dong J, Zhou Y (2022). Tetrahedron Lett.

[18] Panda S P, Hota S K, Dash R, Roy L, Murarka S (2023). Org Lett.

[19] Wang J, Song Q, He X, Ma C, Jiang Y, Fan J (2022). New J Chem.

[20] Shao Z, Zhou Q, Wang J, Tang R, Shen Y (2021). Chin J Org Chem.

[21] Liu C, Shen N, Shang R (2021). Org Chem Front.

[22] Wang G-Z, Fu M-C, Zhao B, Shang R (2021). Sci China: Chem.

[23] Prier C K, Rankic D A, MacMillan D W C (2013). Chem Rev.

[24] Li R-H, Zhao Y-L, Shang Q-K, Geng Y, Wang X-L, Su Z-M, Li G-F, Guan W (2021). ACS Catal.

[25] Yi H, Zhang G, Wang H, Huang Z, Wang J, Singh A K, Lei A (2017). Chem Rev.

[26] Murray P R D, Leibler I N-M, Hell S M, Villalona E, Doyle A G, Knowles R R (2022). ACS Catal.

[27] Liu X-J, Zhou S-Y, Xiao Y, Sun Q, Lu X, Li Y, Li J-H (2021). Org Lett.

[28] Ibarra I A, Islas-Jácome A, González-Zamora E (2018). Org Biomol Chem.

[29] Liao J, Yang X, Ouyang L, Lai Y, Huang J, Luo R (2021). Org Chem Front.

[30] Bur S K, Padwa A (2007). Adv Heterocycl Chem.

[31] Lu L-Q, Chen J-R, Xiao W-J (2012). Acc Chem Res.

[32] Jiao M-J, Liu D, Hu X-Q, Xu P-F (2019). Org Chem Front.

[33] Liu H-Y, Lu Y, Li Y, Li J-H (2020). Org Lett.

[34] Zhang W-K, Li J-Z, Zhang C-C, Zhang J, Zheng Y-N, Hu Y, Li T, Wei W-T (2022). Eur J Org Chem.

[35] Fan X, Liu H, Ma S, Wang F, Yang J, Li D (2022). Tetrahedron.

[36] Liu D, Zhao Y, Patureau F W (2023). Beilstein J Org Chem.

[37] Wadekar K, Aswale S, Yatham V R (2020). RSC Adv.

[38] Liu Y, Sui J-L, Yu W-Q, Xiong B-Q, Tang K-W, Zhong L-J (2023). J Org Chem.

[39] Tafesh A M, Weiguny J (1996). Chem Rev.

[40] Formenti D, Ferretti F, Scharnagl F K, Beller M (2019). Chem Rev.

[41] Song J, Huang Z-F, Pan L, Li K, Zhang X, Wang L, Zou J-J (2018). Appl Catal, B.

[42] Guo Q, Ma Z, Zhou C, Ren Z, Yang X (2019). Chem Rev.

[43] Xiao Q, Sarina S, Waclawik E R, Jia J, Chang J, Riches J D, Wu H, Zheng Z, Zhu H (2016). ACS Catal.

[44] Tsutsumi K, Uchikawa F, Sakai K, Tabata K (2016). ACS Catal.

[45] Qu Z, Chen X, Zhong S, Deng G-J, Huang H (2021). Org Lett.

[46] Fu M-C, Wang J-X, Shang R (2020). Org Lett.

[47] Sheng H, Liu Q, Su X-D, Lu Y, Wang Z-X, Chen X-Y (2020). Org Lett.

[48] Liang T, Neumann C N, Ritter T (2013). Angew Chem, Int Ed.

[49] Reichel M, Karaghiosoff K (2020). Angew Chem, Int Ed.

[50] Liu Q, Lu Y, Sheng H, Zhang C-S, Su X-D, Wang Z-X, Chen X-Y (2021). Angew Chem, Int Ed.

[51] Hou T, Peng H, Xin Y, Wang S, Zhu W, Chen L, Yao Y, Zhang W, Liang S, Wang L (2020). ACS Catal.

[52] Yao W, Bazan-Bergamino E A, Ngai M-Y (2022). ChemCatChem.

